# Age-related autoimmunity

**DOI:** 10.1186/1741-7015-11-94

**Published:** 2013-04-04

**Authors:** Zahava Vadasz, Tharwat Haj, Aharon Kessel, Elias Toubi

**Affiliations:** 1 Division of Allergy and Clinical Immunology, Bnai-Zion Medical Center, Golomb Street 47, Haifa, 31048, Israel

**Keywords:** Aging, Autoimmunity, Cancer, Sepsis, T-regulatory cells

## Abstract

Older persons have higher autoimmunity but a lower prevalence of autoimmune diseases. A possible explanation for this is the expansion of many protective regulatory mechanisms highly characteristic in the elderly. Of note is the higher production of peripheral T-regulatory cells.

The frequent development of autoimmunity in the elderly was suggested to take place in part due to the selection of T cells with increased affinity to self-antigens or to latent viruses. These cells were shown to have a greater ability to be pro-inflammatory, thereby amplifying autoimmunity. During aging, thymic T-regulatory cell output decreases in association with the loss of thymic capacity to generate new T cells. However, to balance the above mentioned autoimmunity and prevent the development of autoimmune diseases, there is an age-related increase in peripheral CD4+ CD25highFoxP3+ T-regulatory cells. It remains unclear whether this is an age-related immune dysfunction or a defense response. Whatever the reason, the expansion of T-regulatory cells requires payment in terms of an increased incidence of cancer and higher susceptibility to infections.

## Introduction

Over the course of the human life, age-related diseases develop because of the failure of genetic traits to remain beneficial, as they were in younger years when they aided in successful reproduction. Longevity is correlated with optimal natural immunity. Immunosenescence (aging of the immune system) is continuously influenced by chronic antigenic stimulation, such as infections. This explains why the probability of a long lifespan is improved in an environment of reduced pathogen burden. In the presence of low pathogen burden one can expect a balanced state of immune responses and alter the chances of having advanced inflammatory responses [1].

In studies of aging, it has been noticed that the capacity to cope with a variety of infections reduces over time, a status that was identified as ‘inflamm-aging’. The persistence of inflammatory stimuli over time serves as a ‘first hit’, increasing susceptibility to age-related diseases. A ‘second hit’, the absence of robust beneficial gene variants, is crucial in the development of advanced organ-specific age-related diseases [2].

Although inflammation (via pro-inflammatory cytokines and acute phase proteins) is important for preventing or neutralizing dangerous infectious agents in young people, it becomes a meaningful stress leading to altered immunoregulatory and/or unbalanced responses in aged individuals. The later development of continuous organ damage in the presence of altered or unbalanced immune responses is responsible for the development of many age-related diseases, including cardiovascular [3,4]. Among other diseases that are age related are the increased prevalence of autoimmunity, autoantibodies and cancers, and an increased susceptibility to bacterial and viral infections. Here, we will focus on some of these issues in relation to aging.

### Autoantibodies and aging

In one of the first studies dealing with the prevalence of non-organ-specific antibodies in the elderly, it was found that rheumatoid factor, antinuclear antibodies and anti-cardiolipin antibodies were detected in 14%, 31% and 51% (respectively) of healthy individuals over 80 years old, in comparison to not more than 2% in the non-elderly population [5]. Other studies have reported on the higher prevalence of both organ- and non-organ-specific autoantibodies among healthy centenarians (age range 101 to 106 years) when compared with prevalence among younger individuals (age range 26 to 60 years). The main increase was noticed in autoantibodies such as antinuclear antibodies, anti-cardiolipin antibodies and anti-thyroid antibodies. This increase in autoantibodies was suggested to be the result of a damaged tissue process and high exposure to apoptotic cells rather than an autoimmune response [6]. These facts were further established when various autoantibodies such as rheumatoid factor, antinuclear antibodies, anti-cardiolipin antibodies, antineutrophil cytoplasmic antibodies and others were assessed in 276 people who celebrated their 100^th^ birthday. Among these, 79% had at least one of the above mentioned antibodies. In this study also, the high level of antibodies did not reflect a similar high level of full-blown autoimmune disease [7].

The mechanisms and the meaning of autoimmunity during aging is not clear. However, it looks as though autoimmunity is only a reflection of the advanced organ damage that takes place during aging and the resulting immune response.

#### Autoimmune diseases in the elderly

In contrast to the frequent prevalence of autoantibodies in the elderly, autoimmune diseases are rare. When they exist, they are mild and well controlled with moderate immunomodulatory therapies. When systemic lupus erythematosus (SLE) was assessed in individuals over 65 years of age, the incidence of late-onset SLE ranged between 12% and 18% and the course of the disease was found to be milder. Skin manifestations, photosensitivity, arthritis and nephritis were rarely reported. However, lung involvement and Sjogren’s syndrome were observed more frequently. In patients with late-onset SLE, one can observe higher prevalence of autoantibodies such as rheumatoid factor, anti-Ro and anti-cardiolipin antibodies but a lower occurrence of hypocomplementemia [8]. A possible explanation for this higher autoimmunity but lower or milder autoimmune diseases is the expansion of many protective regulatory mechanisms highly characteristic in the elderly. Of note is the higher production of protective natural immunoglobulin M autoantibodies, such as immunoglobulin M anti-cardiolipin and immunoglobulin M anti-double stranded DNA antibodies. All these autoantibodies have been reported to play a role in preventing the development of severe SLE and are higher in patients without renal disease [9].

### T-regulatory cells and aging

The frequent development of autoimmunity in the elderly may occur in part due to the selection of T cells with increased affinity to self-antigens or to latent viruses. These T cells have been shown to have a greater ability to be pro-inflammatory, thereby amplifying autoimmunity [10]. During aging, the output of thymic T-regulatory cells (Tregs) decreases in association with the loss of thymic capacity to generate new T cells. However, to balance the above and prevent the development of autoimmune diseases, there is an age-related increase in peripheral generation of CD4+ CD25highFoxP3+ Tregs. It remains unclear whether this is an immune dysfunction or a defense response aiming to balance the increase in autoimmunity. Whatever the reason, the expansion of Tregs requires payment in terms of an increased incidence of cancer and higher susceptibility to infections [11].

#### T-regulatory cells and cancer

Increased autoimmunity during aging has been explained by many to be the result of Tregs, though expanded, failing to suppress auto-reactive T cells (in response to enhanced apoptosis). Although young and aged CD4+ Tregs equally suppressed interferon-γ + T cells in a mouse model, aged Tregs failed to restrain IL-17+ T cells during inflammation, suggesting a chronic inflammation-related defect in aged Tregs. The aged Tregs expressed reduced STAT3 activation, a defect that was found to be in association with poor IL-17-producing T cell restraint, which may contribute to the development of autoimmunity in the elderly [12]. By contrast, many studies have shown that Tregs (both in animal models and humans) are expanded in the elderly. This results in increased suppression of T cell immune responses and the prevention of autoimmune diseases, but increases susceptibility to infectious diseases and cancer, which become the leading causes of morbidity and mortality in the elderly [13].

The role of immunosuppressive Tregs in tumor immune evasion and metastatic spread is well established. Therefore, one may assume that changes in numbers or function of Tregs could lead to a higher incidence of tumors in the elderly. Many studies have been designed to assess this relationship. In one of these, the percentage of and changes in FoxP3 expression in CD4 + CD25highCD127low were analyzed in older people in relation to the development of non-small cell lung cancer. The percentage of peripheral Tregs and the expression of FoxP3 mRNA were significantly increased in elderly patients with non-small cell lung cancer compared with healthy elderly and young individuals. The percentage of Tregs and the expression of FoxP3 mRNA were closely associated with tumor node metastasis staging in elderly patients with lung cancer [14].

#### Tregs and sepsis

Inducible Tregs are important in keeping peripheral tolerance and in preventing CD4+ T cells from responding to T cell receptor stimulation and entering the cell cycle. One of these subsets is CD8 + CD45RA + C-C chemokine receptor 7 (CCR7) + Foxp3 T cells, the suppressive activity of which is independent of IL-10 and relies on interference with very early steps of the T cell receptor signaling cascade. The inducibility of CD8 + CCR7+ Tregs was shown to be age related and their number in individuals older than 60 years was much lower than in younger people. Loss of CD8 + CCR7+ Tregs in the elderly host is of relevance in the aging immune system because immunosenescence is associated with a state of chronic smoldering inflammation [15]. The status of Tregs in respect to the beneficial immune response during sepsis has also been assessed. In elderly patients, an increased percentage of circulating Tregs significantly correlated with a decreased lymphoproliferative response. In a murine model of sepsis mimicking these observations, the *ex vivo* downregulation of FoxP3 expression using siRNA was associated with a restoration of this response [16].

### Final comments

**Figure 1:**
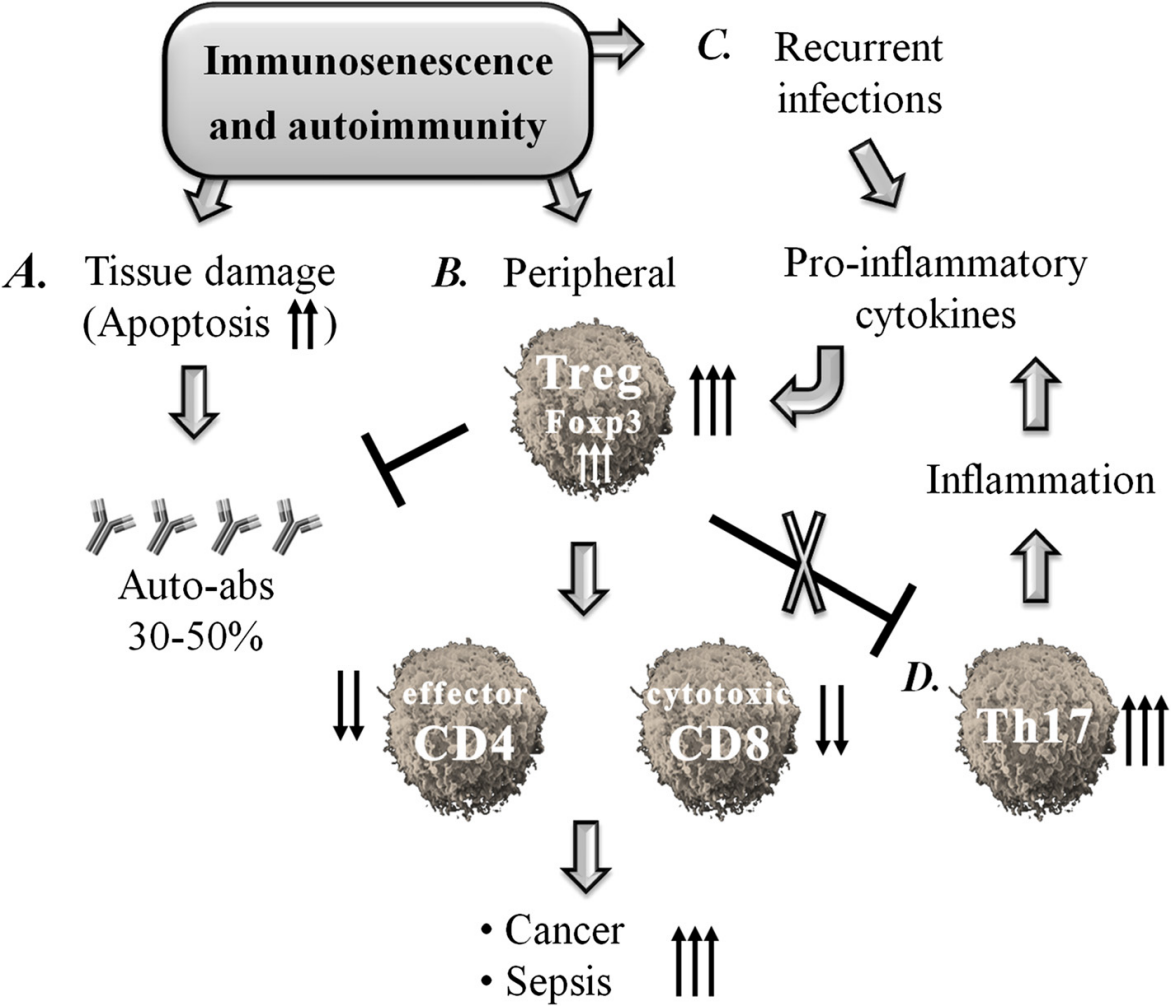
**Increased T-regulatory cell function in the elderly balances increased autoimmunity but increases the incidence of cancer and sepsis. (A)** Autoantibodies are frequent in aged individuals because of increased tissue damage and apoptosis. **(B)** Aiming to balance this increased autoimmunity, peripheral Tregs become enhanced, suppressing both CD4 and CD8 T cell function, allowing the development of cancers and increasing susceptibility to infections. **(C)** Recurrent viral and bacterial infections stimulate pro-inflammatory cytokines, which are further stimulated by this expansion of Tregs. **(D)** Treg expansion in the elderly is followed by the increase of T-helper 17 cells and the persistence of chronic inflammation.

In the elderly, autoimmunity is balanced by the immune response expanding the number of peripheral Tregs, which prevents full-blown autoimmune diseases. This natural process must remain tuned and reasonable, otherwise CD4+ T effector cells or CD8 cytotoxic T cells will be suppressed, allowing the development of cancers and sepsis (see Figure 1). One of the future goals is to determine how to affect this tuning, namely, how to immunomodulate Tregs in the elderly.

## Abbreviations

CCR7: C-C chemokine 7; IL: Interleukin; SLE: Systemic lupus erythematosus; Tregs: T-regulatory cells.

## Competing interests

The authors declare that they have no competing interests.

## Authors’ information

ZV is a senior physician in the Division of Allergy and Clinical Immunology and PhD in Biology of Cancer. She is active in treating patients with autoimmune diseases. She is supervising some research projects in the field of autoimmunity. TH recently received her PhD degree in Immunology. She is now a senior researcher in the field of autoimmunity. AK is a senior physician in the division of Allergy and Clinical Immunology and leads the research activity of food allergy, atopic dermatitis and asthma as well as running research projects in various fields of autoimmunity. ET is head of Division of Allergy and Clinical Immunology and is responsible for all research activities of the division.

## Pre-publication history

The pre-publication history for this paper can be accessed here:

http://www.biomedcentral.com/1741-7015/11/94/prepub
